# Southern Medical College, Atlanta

**Published:** 1882-09-20

**Authors:** 


### *SOUTHERN MEDICAL COLLEGE, ATLANTA.*

This Institution opens the present year on the 5th of October, giving greater length of session than heretofore. The school is now fully equipped with both Hospital and Clinical advantages. The curriculum of study embraces all departments, and the Faculty composed of active and zealous men in the profession, experienced as teachers, and well up with all the modern advances in medical science.

THE Eighth Annual meeting of the Tri-State Medical Society will be held at Terre Haute, Indiana, September 26th, 27th and 28th, 1882, with the following officers:

President—J. M. Holloway, Louisville, Ky.
Secretary—G. W. Burton, Mitchell, Indiana.
Treasurer—F. W. Beard, Vincennes, Indiana.
J. E. Link, Terre Haute, Ind., Chairman of Committee of Arrangements.
Wm. Porter, St. Louis, Missouri, Chairman of Committee of Programme.

